# The 6 degrees-of-freedom range of motion of the L1–S1 vertebrae in young and middle-aged asymptomatic people

**DOI:** 10.3389/fsurg.2022.1002133

**Published:** 2022-10-26

**Authors:** Fei Xu, Siyu Zhou, Zhuofu Li, Shuai Jiang, Ze Chen, Zhuoran Sun, Weishi Li

**Affiliations:** ^1^ Orthopaedic Department, Peking University Third Hospital, Beijing, China; ^2^ Peking University Health Science Center, Beijing, China; ^3^ Beijing Key Laboratory of Spinal Disease Research, Beijing, China; ^4^ Engineering Research Center of Bone and Joint Precision Medicine, Ministry of Education, Beijing, China

**Keywords:** range of motion, vertebrae, 6 degrees of freedom, young and middle-age, coupled motion

## Abstract

**Study design:**

Controlled laboratory study.

**Objective:**

To determine the 6 degrees of freedom of lumbar vertebra *in vivo* during different functional activities in young and middle-aged asymptomatic subjects.

**Methods:**

A total of 26 asymptomatic subjects (M/F, 15/11; age, 20–55 years) were recruited in this study. They were divided into two groups: young group (number: 14; age: 20–30 years old) and middle-aged group (number: 12; age: 45–55 years old). The lumbar segment of each subject was scanned by computed tomography for the construction of three-dimensional (3D) models of the vertebra from L1 to S1. The lumbar spine was imaged by using a dual fluoroscopic system when the subjects performed different trunk postures. The 3D models of vertebrae were matched to two fluoroscopic images simultaneously in software. The range of motion (ROM) of vertebrae in the young and middle-aged groups was compared by using multiway analysis of variance, respectively.

**Results:**

During the supine to the upright posture, vertebral rotation of the L1–S1 occurred mainly around the mediolateral axis (mean: 3.9 ± 2.9°). Along the mediolateral axis, vertebral translation was significantly lower at L1–2 (7.7 ± 2.4 mm) and L2–3 (8.0 ± 3.5 mm) than at L3–4 (1.6 ± 1.2 mm), L4–5 (3.3 ± 2.6 mm), and L5–S1 (2.6 ± 1.9 mm). At the L4–5 level, the young group had a higher rotational ROM than the middle-aged group around all three axes during left–right bending. Along the anteroposterior axis, the young group had a lower translational ROM at L4–5 than the middle-aged group during left–right bending (4.6 ± 3.3 vs. 7.6 ± 4.8 mm; *P* < 0.05). At L5–S1, the young group had a lower translational ROM than the middle-aged group during flexion–extension, left–right bending, and left–right torsion.

**Conclusion:**

This study explored the lumbar vertebral ROM at L1–S1 during different functional postures in both young and middle-aged volunteers. There were higher coupled translations at L3–4 and L4–5 than at the upper lumbar segments during supine to upright. The vertebral rotation decreased with age. In addition, the older subjects had a higher anteroposterior translation at the L4–5 segment and higher mediolateral translation at the L5–S1 segment than the young group. These data might provide basic data to be compared with spinal pathology.

## Introduction

Lumbar degenerative disc disease (DDD) always occurs at the lower lumbar levels, and the incidence of various pathological changes is segment-dependent. For example, lumbar degenerative spondylolisthesis is more likely to occur at L4–5 (1, 2) and more lumbar disc herniation is observed at L5–S1 (3). Vertebral segment motion is important for maintaining spinal stability. Altered vertebral motion is known to change spinal biomechanics, which is related to spinal pathology (4–8).

Most studies have focused on the range of motion (ROM) of the vertebrae *in vitro* instead of *in vivo* (9–14). Some *in vivo* experiments have reported the motion of the lumbar segments using imaging techniques to capture the lumbar vertebral positions in different postures (5, 15, 16). Recently, a three-dimensional (3D) fluoroscopic imaging technique has been applied to investigate the degree of freedom (DOF) of the lumbar vertebrae during various weight-bearing end-range postures and dynamic motion of the trunk in subjects aged over 40 years (17–19). However, there are still no data on the comparison of L1–S1 vertebral kinematics between different age groups. The 6DOF of the L1–S1 vertebral segments *in vivo* from the supine to the upright posture has not been previously reported.

This study aimed to determine the 6DOF of the L1–S1 vertebral segments in young and middle-aged asymptomatic human subjects during supine to upright, flexion, extension, left–right bending, and left–right torsion. We hypothesized that the segmental kinematics of primary motion and coupled motion was different in the young and middle-aged groups and that the segmental kinematics was different at different levels from a supine to an upright posture.

## Materials and methods

### Characters of participants

In this study, we enrolled 26 asymptomatic subjects aged between 20 and 55 years. There were 15 males and 11 females. Patients were divided into two groups: 14 subjects in the young group [median, 24.9 ± 2.1 (range, 20–30) years] and 12 subjects in the middle-aged group [median, 52.1 ± 3.2 (range, 45–55) years]. The institutional review board of the authors’ hospital approved the experimental design before starting the study. Participants were evaluated for the presence or absence of lower back pain and other spinal disorders. The exclusion criteria were as follows: (1) current or prior serious back pain; (2) history of spinal surgery; (3) diagnosis of disease or anatomical anomaly in the spine; (4) prior radiation within a year; and (5) pregnancy. Before testing, each volunteer signed an informed consent form.

### 3D anatomical vertebral model and dual fluoroscopic imaging system

**Figure 1:**
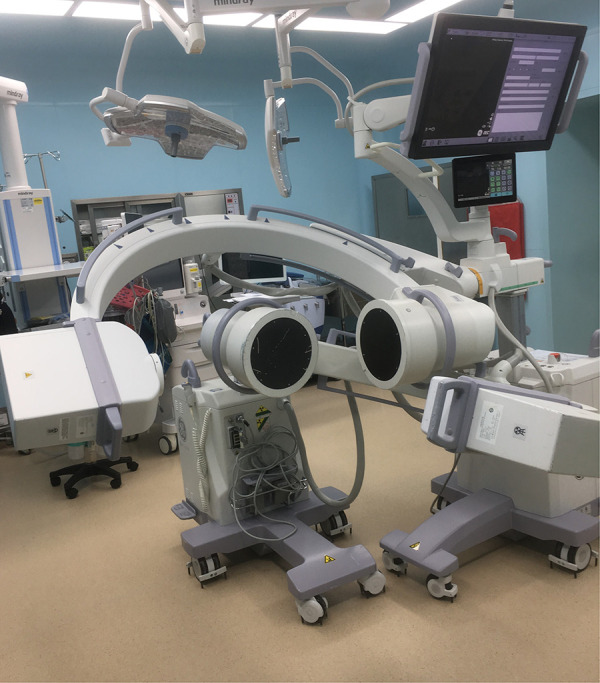
Two fluoroscopes were positioned with their image intensifiers kept perpendicular to each other.

**Figure 2:**
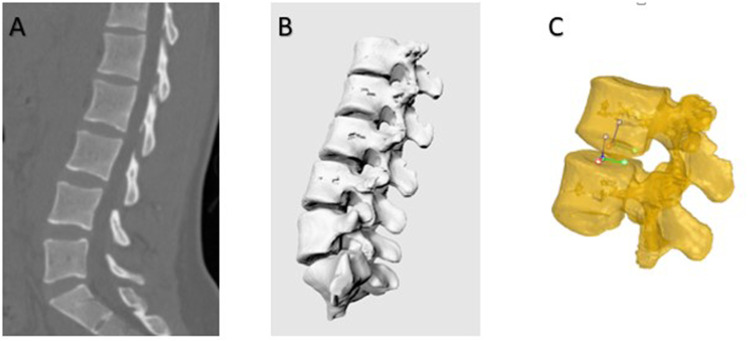
(**A**) A typical CT scan of a human lumbar spine in the sagittal plane. (**B**) Three-dimensional anatomic vertebral model constructed from computed tomography. (**C**) Anatomic coordinate systems were established at the endplates to measure the kinematics of the vertebrae.

**Figure 3:**
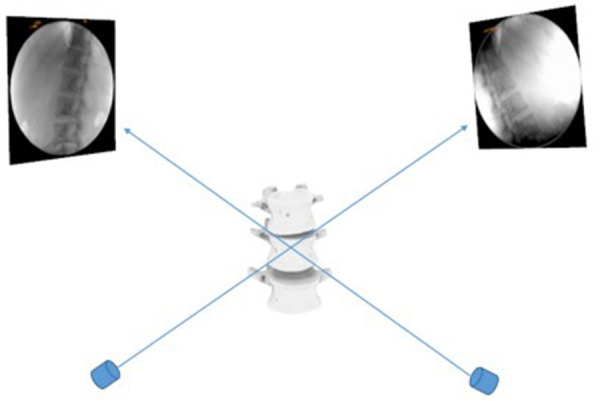
Virtual reproduction of the dual fluoroscopic system and the vertebral positions.

Each subject underwent a computed tomography (CT) scan (Sensation; Siemens, Erlangen, Germany). Parallel digital images with a thickness of 0.625 mm without a gap were obtained. Following CT scanning, two fluoroscopes (BV Pulsera, Phillips, Bothell, WA, United States) were positioned, with their image intensifiers kept perpendicular to each other (Figure 1). Accordingly, we captured images of the lumbar spine simultaneously in two different directions: upright posture, 45° flexion of the trunk, maximal extension, maximal left–right bending, and maximal left–right twisting. The patients were in different functional postures for both fluoroscopes. The CT images of the spinal segments were imported into Mimics version 21.0 (Materialize, Leuven, Belgium) to construct 3D anatomical vertebral models of the L1, L2, L3, L4, L5, and S1 segments. The 3D models of the vertebrae were subsequently created from contour lines (Figures 2A,B). Thereafter, these models were imported into a virtual dual orthogonal environment (Rhinoceros, Robert McNeel / Associates, Seattle, WA, United States). The repeatability of the method in reproducing *in vivo* human spine 6DOF kinematics was <0.3 mm in translation and <0.7° in orientation (20). The positions of the vertebrae at different postures of the trunk under physiological loads were reproduced in Rhinoceros software using the 3D models of the vertebrae and orthogonal fluoroscopic images. The models could be independently translated and rotated in 6DOF until their outlines matched the two orthogonal fluoroscopic images simultaneously (Figure 3) (20).

After reproducing the *in vivo* vertebral positions in the dual fluoroscopic image system, the relative motion of the vertebrae was analyzed using the right-hand Cartesian coordinate systems constructed at the geometric center of the vertebral endplates (Figure 2C). The x-axis was set in the frontal plane to represent the mediolateral direction and pointed to the left. The y-axis was set in the sagittal plane and pointed posteriorly. The z-axis was set perpendicular to the x-y plane representing the cephalad–caudad direction and pointed cranially. The relative motion of the proximal endplates with respect to the distal endplates was calculated at five levels: L1–2, L2–3, L3–4, L4–5, and L5–S1. The ROM data included both primary rotations and translation, coupled translations, and rotations in all 6DOFs.

### Statistical analysis

A two-way repeated measures ANOVA was used to compare the ROM of the vertebrae at the L1–L2, L2–L3, L3–L4, L4–L5, and L5–S1 levels. Kinematics was the dependent variable, and vertebral level and activity were the independent variables. A *P*-value <0.05 was considered statistically significant. Another multiway analysis of variance was used to compare the kinematics between the young and the middle-aged subjects. The participant group was the categorical factor, and the levels and activities were the independent variables. A Newman–Keuls *post hoc* test was performed when a statistically significant difference was detected. Statistical analysis was performed using SPSS version 23 (IBM Corp.) and Prism 9 software version 5.01 (GraphPad Software Inc., CA, United States).

## Results

### Rotated and translated vertebral motions from supine to upright posture

**Figure 4:**
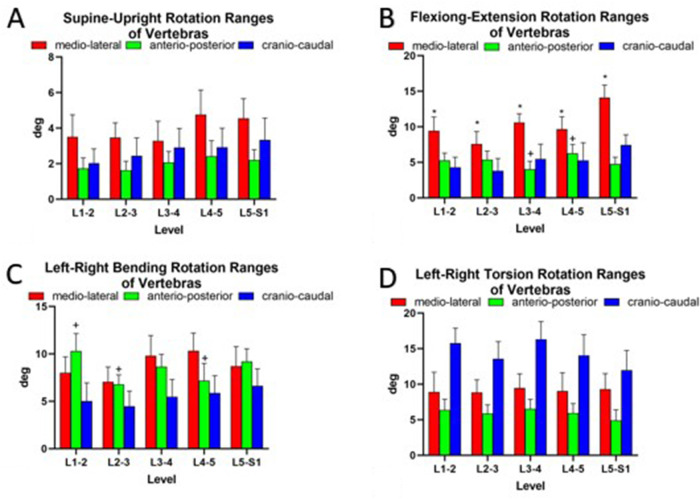
Ranges of vertebral rotations of asymptotic volunteers (**A**) standing up and along three principal axes under (**B**) flexion–extension, (**C**) bending, and (**D**) torsion of the trunk. The symbols (*, +, x, #, −) represent statistical significance on between-level comparison (*P* < 0.05).

**Figure 5:**
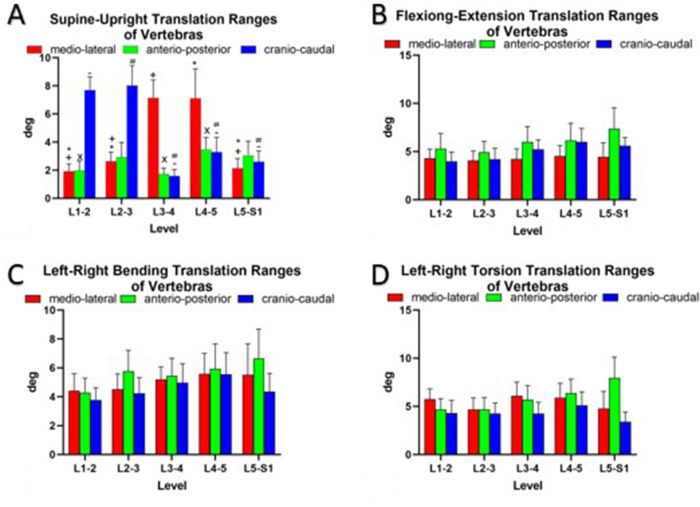
Ranges of vertebral translations of asymptotic volunteers (**A**) standing up and along three principal axes under (**B**) flexion–extension, (**C**) bending, and (**D**) torsion of the trunk. The symbols (*, +, x, #, −) represent statistical significance on between-level comparison (*P* < 0.05).

From the supine posture to the upright posture, vertebral rotation occurred mainly around the mediolateral axis (mean: 3.9 ± 2.9°) (Figure 4A). The vertebral rotations were not significantly different between different levels around the mediolateral axis (L1–2, 3.6° ± 3.0°; L2–3, 3.5° ± 2.0°; L3–4, 3.3° ± 2.7°; L4–5, 4.8° ± 3.4°; and L5–S1, 4.6° ± 2.7°) (Figure 4A). There were coupled translations in all three directions. The coupled translations along the craniocaudal axis were significantly higher at L1–2 (7.7 ± 2.4 mm) and L2–3 (8.0 ± 3.5 mm) than at L3–4 (1.6 ± 1.2 mm), L4–5 (3.3 ± 2.6 mm), and L5–S1 (2.6 ± 1.9 mm). Along the mediolateral axis, vertebral translations were significantly lower at L1–2 (1.9 ± 1.3 mm) and L2–3 (2.6 ± 1.6 mm) than at L3–4 (7.2 ± 3.1 mm) and L4–5 (7.1 ± 5.2 mm) (Figure 5A).

### Rotational and translational vertebral motions during flexion–extension, bending, and torsion postures

During flexion–extension positions of the trunk, the predominant rotational axis was around the mediolateral axis (mean, 10.3° ± 4.7°) (Figure 4B). The rotational ROM around the mediolateral axis at L5–S1 (14.1° ± 4.3°) was higher than that at the other levels (L1–2, 9.5° ± 4.8°; L2–3, 7.6° ± 4.4°; L3–4, 10.6° ± 3.0°; and L4–5, 9.7° ± 4.3°; *P* < 0.05, all) (Figure 4B). During the left–right bending of the trunk, a combination of rotation in all three orthogonal directions was observed (Figure 4C). Around the anteroposterior axis, vertebral rotation was higher at L1–2 (10.3 ± 4.5°) than at L2–3 and L4–5 (6.8° ± 2.5° and 7.2° ± 4.5°, respectively; *P* < 0.05) (Figure 4C). Left–right torsion of the trunk was achieved by coupled rotation in different directions (Figure 4D). The principal rotation was around the craniocaudal axis. Around the craniocaudal axis, there was no significant difference between different levels (*P* > 0.05) (Figure 4D). The coupled translations in the three directions were not significantly different at the different levels during flexion–extension, left–right bending, and left–right torsion postures (Figure 5).

### Comparison of young and middle-aged healthy participants

From the supine posture to the upright posture, around the anteroposterior axis, young healthy participants had a higher vertebral ROM than the middle-aged group at L4–5 (*P* < 0.05) (Table 1). As for vertebral translation, there was no significant difference between the young and the middle-aged groups (*P* > 0.05) (Table 2).

**Table 1:** Comparison of rotation ranges (°) of vertebras between young and middle-aged participants.

	L1–L2			L2–L3			L3–L4			L4–L5			L5–S1		
	ML	AP	CC	ML	AP	CC	ML	AP	CC	ML	AP	CC	ML	AP	CC
Supine-standing															
Young	3.67 ± 3.78	2.17 ± 1.69	1.69 ± 1.66	3.57 ± 1.95	1.20 ± 0.91	2.03 ± 2.09	3.64 ± 3.06	1.76 ± 1.44	3.39 ± 3.10	4.31 ± 2.91	3.03 ± 2.51	3.03 ± 2.97	4.25 ± 3.08	1.95 ± 1.29	3.50 ± 3.37
Old	3.32 ± 2.14	1.23 ± 0.96	2.41 ± 2.34	3.35 ± 2.23	2.13 ± 1.43	2.93 ± 2.91	2.87 ± 2.35	2.41 ± 1.66	2.34 ± 2.05	5.31 ± 3.95	1.68 ± 1.60	2.78 ± 2.42	4.94 ± 2.14	2.51 ± 1.63	3.12 ± 2.54
*P*	0.762	0.143	0.464	0.846	0.146	0.359	0.500	0.310	0.284	0.382	0.035*	0.800	0.546	0.382	0.716
Flexion–extension															
Young	9.54 ± 5.47	5.33 ± 2.45	4.56 ± 4.25	7.92 ± 5.36	6.04 ± 3.13	4.43 ± 5.12	11.21 ± 2.59	4.13 ± 3.22	6.85 ± 6.26	10.08 ± 4.34	6.44 ± 2.80	6.70 ± 6.75	14.27 ± 4.33	4.46 ± 2.42	6.51 ± 3.04
Old	9.33 ± 4.01	5.22 ± 2.66	3.99 ± 2.64	7.12 ± 3.11	4.54 ± 2.97	3.06 ± 3.26	9.91 ± 3.44	3.91 ± 2.39	3.82 ± 3.18	9.18 ± 4.47	6.10 ± 3.53	3.59 ± 5.18	13.93 ± 4.35	5.17 ± 2.22	8.59 ± 3.86
*P*	0.901	0.922	0.769	0.633	0.195	0.475	0.438	0.849	0.118	0.592	0.767	0.109	0.844	0.531	0.269
Left–right bend															
Young	8.42 ± 4.59	11.34 ± 4.64	5.37 ± 4.99	6.01 ± 3.29	7.48 ± 2.39	4.82 ± 4.66	9.86 ± 5.58	8.79 ± 2.37	6.17 ± 4.20	12.06 ± 4.09	8.85 ± 4.79	7.95 ± 4.69	7.62 ± 4.57	8.55 ± 1.92	5.66 ± 4.26
Old	7.53 ± 3.76	9.11 ± 4.28	4.60 ± 4.70	8.25 ± 4.36	5.96 ± 2.54	4.05 ± 3.23	9.78 ± 5.07	8.50 ± 4.14	4.68 ± 4.93	8.34 ± 4.50	5.24 ± 3.44	3.47 ± 2.79	10.07 ± 5.42	10.06 ± 4.31	7.85 ± 4.38
*P*	0.614	0.129	0.654	0.205	0.300	0.655	0.963	0.844	0.389	0.037*	0.015	0.011*	0.183	0.303	0.214
Left–right torsion															
Young	9.05 ± 6.47	6.95 ± 4.12	15.17 ± 4.14	9.86 ± 4.67	7.06 ± 3.34	15.55 ± 6.26	8.34 ± 4.70	7.05 ± 2.83	16.88 ± 6.31	9.74 ± 5.63	6.93 ± 3.40	15.98 ± 6.84	8.63 ± 5.40	4.42 ± 3.74	8.03 ± 2.87
Old	8.74 ± 7.66	5.71 ± 3.30	16.48 ± 6.37	7.61 ± 3.89	4.47 ± 2.00	11.30 ± 4.91	10.74 ± 5.10	5.93 ± 3.87	15.64 ± 6.29	8.18 ± 7.44	4.77 ± 2.93	11.79 ± 7.27	10.18 ± 5.27	5.49 ± 3.67	17.05 ± 6.68
*P*	0.892	0.341	0.587	0.327	0.049*	0.080	0.295	0.392	0.607	0.496	0.098	0.085	0.501	0.436	<0.001**

Rotation around the axis: ML, AP, and CC.

The values were presented as mean ± SD in degree.

AP, anteroposterior; CC, craniocaudal; ML, mediolateral.

*<0.05; **<0.01.

**Table 2:** Comparison of translation ranges (mm) of vertebras between young and middle-aged participants.

	L1–L2			L2–L3			L3–L4			L4–L5			L5–S1		
	ML	AP	CC	ML	AP	CC	ML	AP	CC	ML	AP	CC	ML	AP	CC
Supine-standing															
Young	1.57 ± 1.22	2.20 ± 1.91	8.41 ± 2.25	2.37 ± 1.2	2.50 ± 2.23	8.15 ± 3.77	1.30 ± 0.67	3.16 ± 1.57	2.42 ± 1.71	2.14 ± 2.14	2.76 ± 1.99	2.51 ± 2.01	1.63 ± 1.07	2.94 ± 1.76	2.35 ± 1.48
Old	2.32 ± 1.39	1.73 ± 1.39	6.85 ± 2.30	2.95 ± 1.99	3.43 ± 2.99	7.88 ± 3.32	1.58 ± 1.17	2.31 ± 1.52	1.80 ± 1.20	1.77 ± 1.48	2.36 ± 1.29	3.45 ± 2.10	2.79 ± 2.13	3.14 ± 3.36	2.91 ± 2.28
*P*	0.559	0.545	0.123	0.651	0.226	0.788	0.855	0.421	0.237	0.234	0.097	0.834	0.339	0.807	0.570
Flexion–extension															
Young	4.57 ± 2.90	5.91 ± 4.50	3.91 ± 2.84	3.89 ± 2.80	5.09 ± 2.37	4.74 ± 3.12	3.88 ± 2.57	5.90 ± 3.62	5.77 ± 2.44	4.77 ± 3.50	6.13 ± 4.31	6.33 ± 4.04	3.26 ± 1.41	5.55 ± 2.02	5.60 ± 1.74
Old	3.97 ± 1.69	4.58 ± 3.16	4.05 ± 1.90	4.28 ± 2.03	4.77 ± 3.31	3.58 ± 2.44	4.61 ± 2.78	6.12 ± 4.45	4.59 ± 2.40	4.26 ± 1.39	6.15 ± 4.75	5.63 ± 2.70	5.99 ± 4.77	9.68 ± 7.18	5.58 ± 2.55
*P*	0.558	0.386	0.899	0.699	0.832	0.301	0.473	0.886	0.293	0.618	0.991	0.533	0.015*	0.014*	0.988
Left–right bend															
Young	3.60 ± 2.80	3.49 ± 2.20	2.94 ± 1.72	3.45 ± 1.29	4.20 ± 3.56	4.84 ± 3.03	4.85 ± 2.31	6.01 ± 3.15	4.90 ± 3.99	6.15 ± 4.41	4.56 ± 3.27	5.00 ± 2.51	2.98 ± 1.12	6.24 ± 4.12	3.89 ± 3.16
Old	5.35 ± 2.95	5.22 ± 2.51	4.71 ± 2.22	5.77 ± 3.25	7.58 ± 2.82	3.53 ± 2.12	5.59 ± 2.04	4.79 ± 2.87	5.04 ± 2.41	4.93 ± 2.15	7.55 ± 4.83	6.20 ± 4.79	8.76 ± 6.56	7.19 ± 5.94	4.93 ± 3.08
*P*	0.117	0.176	0.138	0.038*	0.009**	0.272	0.503	0.338	0.908	0.270	0.020*	0.313	<0.001**	0.520	0.395
Left–right torsion															
Young	5.24 ± 1.80	4.58 ± 2.53	3.61 ± 2.31	4.71 ± 2.56	3.67 ± 3.13	3.51 ± 2.76	5.88 ± 3.67	6.59 ± 4.06	5.35 ± 3.13	6.44 ± 4.48	6.27 ± 3.20	4.81 ± 2.94	2.49 ± 1.66	6.93 ± 3.68	3.36 ± 2.59
Old	6.37 ± 3.32	4.78 ± 3.21	5.12 ± 4.01	4.64 ± 3.54	5.85 ± 2.69	5.11 ± 2.57	6.37 ± 3.47	4.68 ± 2.72	2.96 ± 2.17	5.26 ± 2.63	6.52 ± 4.05	5.42 ± 4.17	7.68 ± 4.96	9.25 ± 6.79	3.45 ± 2.40
*P*	0.386	0.879	0.214	0.961	0.090	0.185	0.702	0.138	0.049*	0.362	0.846	0.615	<0.001**	0.125	0.939

Rotation around the axis: ML, AP, and CC.

The values were presented as mean ± SD in mm.

AP, anteroposterior; CC, craniocaudal; ML, mediolateral.

*<0.05; **<0.01.

During the flexion–extension of the trunk, the young healthy participants had higher rotational ROMs than their middle-aged counterparts around the primary mediolateral axis, yet this difference was not statistically different (Table 1). Moreover, along the mediolateral axis and anteroposterior axis, vertebral translations at L5–S1 were higher in the middle-aged group than in the young group (3.3 ± 1.4 vs. 6.0 ± 4.8 mm and 5.6 ± 2.0 vs. 9.7 ± 7.2 mm, respectively; *P* < 0.05, both) (Table 2).

During the left–right bending of the trunk (Table 1), at the L4–5 level, the young group had higher rotational ROMs than the middle-aged group around all three axes (12.1° ± 4.1° vs. 8.3° ± 4.5°, 8.9° ± 4.8° vs. 5.2° ± 3.4°, and 8.0° ± 4.7° vs. 3.5 ± 2.8°, respectively; *P* < 0.05, all). As for the translation along the anteroposterior axis, the young group had a lower ROM than the middle-aged group at L4–5 (4.6 ± 3.3 vs. 7.6 ± 4.8 mm; *P* < 0.05) (Table 2). Along the mediolateral axis, the young group had a lower translational ROM than the middle-aged group at L5–S1 (3.0 ± 1.1 vs. 8.8 ± 6.6 mm; *P* < 0.05) (Table 2).

During trunk torsion, around the anteroposterior axis at L2–3, young volunteers had a higher vertebral rotation than their middle-aged counterparts (7.1° ± 3.3° vs. 4.5° ± 2.0°, *P* < 0.05) (Table 1). As for the translation along the craniocaudal axis, the young group had a higher ROM than the middle-aged group at L3–4 (5.4 ± 3.1 vs. 3.0 ± 2.2 mm; *P* < 0.05). Along the mediolateral axis, the coupled translation at L5–S1 was higher in the middle-aged group than in the young group (7.7 ± 5.0 vs. 2.5 ± 1.7 mm; *P* < 0.05) (Table 2).

## Discussion

In this study, we collected quantitative vertebral kinematics in both young and middle-aged asymptomatic subjects in the supine to upright, flexion–extension, left–right bending, and left–right torsion postures. The data indicated that the coupled translation was different at different levels during the supine to the upright postures. Moreover, during comparison, rotation was higher at L4–5 in the young group than in the middle-aged group. However, the coupled translations in the anteroposterior direction at L4–5 and in the mediolateral direction at L5–S1 in the young group were lower than those in the middle-aged group.

Many studies have reported the segmental ROM of the lumbar spine using various experimental designs. Li et al. (17) reported vertebral kinematics at L2–L5 during different postures in old subjects using combined dual fluoroscopic and magnetic resonance imaging (MRI) techniques. In our study, we also collected vertebral motion at L1–2 and L5–S1. Moreover, we involved the posture from the supine to the upright posture, which has not been studied *in vivo* before (17–19, 21). We also increased the sample size to include young and middle-aged subjects. Pearcy (22) investigated *in vivo* lumbar vertebral motion at approximately 14° at each lumbar level during maximal flexion–extension positions, and Wu et al. (23) also studied lumbar vertebral motion. L4–5 showed the largest anteroposterior translation (2.9 ± 1.5 mm), and L5–S1 showed the largest craniocaudal translation (2.8 ± 0.9 mm) during flexion–extension positions, where the pelvis and hips were limited. Our data showed higher translation at L4–5 and L5–S1 measured without the pelvis under limitation. Pearcy (22) reported larger bending ranges in the upper vertebral segments than in the lower vertebral levels, which was in line with the findings of our study. This could be related to the different anatomic orientations of the facet joints at different levels, as the L1–2 facet was oriented more vertically than L4–5 (24).

Shin et al. (19) studied lumbar vertebral motion during axial rotation with subjects holding a 16-pound dumbbell and found no significant difference in the range of primary axial rotation between different segmental levels, which was in line with the results of our study. In their study, they reported that the ROM of the vertebra was approximately 6° at each segment during left–right twisting, which was lower than that in our study. The reason might be that the subjects were only in a standing weight-bearing posture, and the pelvis was not limited in our study, enlarging the vertebral rotational ROM. Haughton et al. (25) investigated lumbar twisting using MR scans with the subject lying supine and showed an average range of axial rotation between 1.8° and 5.7° at the three vertebral levels. Ochia et al. (26) determined that the upper lumbar motion segments had a greater axial rotation range compared with the lower segments when the upper body was passively rotated to ±50° in the supine posture, which was similar to our result. However, using MRI scanning, Li et al. (17). reported that the ROM of the vertebra was approximately 2° or 3° during left–right torsion. These large discrepancies in the vertebral rotation could be explained by the various loading conditions used in these studies, which were caused by the different experimental setups used. In our study, we included young and middle-aged participants who were younger than those in previous studies (17, 19). Moreover, all participants in our study were Asian. A quantitative comparison between these studies might be difficult, considering the different loading conditions and ages of the participants.

It was reported that coupled axial translational and rotational characteristics were different between the supine and the upright postures in an *in vitro* experiment because of body weight (27). Dehghan-Hamani et al. (28) recently noted that moving from the supine to the upright posture altered not only the translational kinematics (0.25–0.75 mm), but also the rotational kinematics (0.0°–7.0°) by finite-element models. However, few studies have reported vertebral kinematics in the supine to the upright posture *in vivo*. Our data demonstrated that the vertebra primarily rotated around the mediolateral axis (mean, 3.9° ± 2.9°). In addition, along the mediolateral axis, translations at L3–L4 and L4–L5 were higher than those at the upper levels, proving that the lower lumbar disc was mainly influenced by the lateral force. This might be related to the fact that degenerative lumbar scoliosis (DLS) was more likely to occur in the lower lumbar segments than in upper lumbar segments (29, 30). The upper lumbar vertebra had higher craniocaudal translation than the lower segments. This phenomenon induced the discs at L3–4 and L4–5 to have a higher speed of degeneration than those at the upper lumbar segments, which had a worse capability of deformation. Previous studies reported that with increased age, the intervertebral disc would degenerate and reduce the capability of deformation (31, 32).

As for the difference between the young and the middle-aged groups, rotational ROMs were higher in the young group than in the middle-aged group at L4–5, which might be related to the fact that the ROM at L4–5 decreased with the degeneration of the lumbar spine. This might be related to degenerative lumbar diseases such as facet joint osteoarthritis and DLS, which occur mainly at the L4–5 segment (33–40). As for the coupled motion, it was interesting to note that the middle-aged group had a higher coupled slip in the anterior–posterior direction at L4–5 than the young group. However, at L5–S1, the middle-aged group exhibited a higher coupled lateral slip in the mediolateral direction. Wu et al. (23) also found that lumbar motion segments at L4–5 and L5–S1 showed larger anteroposterior and mediolateral translations in older subjects. These changes are related to spinal instability, but the biomechanical mechanisms of the lumbar spine that are related to these kinematic characteristics are unclear. Epidemiological studies have reported that most instances of degenerative spondylolisthesis occur at L4–5 rather than at other levels (1, 38). Degenerative spondylolisthesis is always accompanied by anterior slippage of the proximal vertebral endplate. The higher anteroposterior translation at L4–5 in older subjects might be related to disease development. In addition, the lower lumbar segment, especially the L5–S1 segment, was more likely to cause lumbar disc herniation than other segments do (3, 41). The higher translation in the mediolateral direction at L5–S1 in older subjects might cause more shear force, which could be a biomechanical factor for lumbar disc herniation. The higher lateral translational motion in older subjects might be related to the fact that the facet joints at L5–S1 were oriented more coronally than those at the upper levels (24, 42). It has been reported that fusion extending to the sacrum is more likely to result in pseudarthrosis (43). Future research should focus on lumbar biomechanical mechanisms in patients with DLS and DDD.

Our study has several limitations. First, flexion was approximately 45° from standing to maximal flexion posture to ensure that the targeted lumbar spine was within the field of view, which might reduce the lumbar ROM during flexion. Subsequently, we collected the instantaneous maximal posture without dynamic motion of the vertebra. Data during the process of changing posture were unavailable.

This paper reported basic data on lumbar vertebral ROM at L1–S1 in both young and middle-aged volunteers during the supine to upright (predominant rotation, 3.9° ± 2.9°), flexion–extension (predominant rotation, 10.25° ± 4.66°), left–right bending (predominant rotation, 8.74° ± 4.37°), and left–right torsion postures (predominant rotation, 14.82° ± 6.34°). There were higher coupled translations at L3–4 and L4–5 than at the upper lumbar segments during the supine to the upright posture. Moreover, vertebral rotation decreased, and coupled translation increased in older subjects. Specifically, compared with their younger counterparts, older subjects had a higher anteroposterior translation at the L4–5 segment and higher mediolateral translation at the L5–S1 segment. These findings provide basic data for making comparisons of spinal pathology between age groups. In addition, this study might be useful for contemporary implant design to prevent adjacent segment degeneration and could provide a more accurate guidance for studying the ROM of the trunk during rehabilitation.

## Data Availability

The original contributions presented in the study are included in the article/Supplementary Material, further inquiries can be directed to the corresponding author.
